# Dental Caries Experience and Use of Dental Services among Brazilian Prisoners

**DOI:** 10.3390/ijerph111212118

**Published:** 2014-11-25

**Authors:** Alessandro Leite Cavalcanti, Iris Sant´Anna Araujo Rodrigues, Ingrid Thays de Melo Silveira, Thaliny Batista Sarmento de Oliveira, Magaly Suenya de Almeida Pinto, Alidianne Fabia Cabral Xavier, Ricardo Dias de Castro, Wilton Wilney Nascimento Padilha

**Affiliations:** 1 Post Graduate Program in Public Health, State University of Paraiba, Campina Grande, PB 58429-500, Brazil; E-Mails: iris.odontosp@gmail.com (I.S.A.A.R.); thalinysarmento@yahoo.com.br (T.B.S.O.); maga.enf@hotmail.com (M.S.A.P.); wiltonpadilha@yahoo.com.br (W.W.N.P.); 2 Department of Dentistry, State University of Paraiba, Campina Grande, PB 58429-500, Brazil; E-Mails: ingridthays_melo@hotmail.com (I.T.M.S.); alidianne.fabia@gmail.com (A.F.C.X.); ricardodiasdecastro@yahoo.com.br (R.D.C.)

**Keywords:** dental caries, tooth loss, prisons, prisoners

## Abstract

This ross-sectional study involving 127 male prisoners evaluates the use of dental services and dental caries among Brazilian inmates. Data were collected by interview and clinical examination. Sociodemographic and sentencing information as well as use of dental services, self-reported dental morbidity, self-perception, and oral health impacts were investigated. The mean DMFT index value was 19.72. Of the components, the decayed component showed the highest mean value (11.06 ± 5.37). Statistically significant association was found between DMFTs with values from 22 to 32 and oral health satisfaction (*p* = 0.002), difficulty speaking (*p* = 0.024), shame of talking (*p* = 0.004) and smiling (*p* < 0.001). Regarding the use of dental services, 80% had their last dental appointment less than one year ago, with most visits occurring in prison (80%), with restorative treatment (32%), followed by dental pain (26.4%), being the main reasons for such appointments. Most prisoners used dental services provided by the prison. Although restorative treatment has been the main reason for the use of dental services, “decayed” and “missing” components contributed to the high mean DMFT index.

## 1. Introduction

Given the precarious conditions of confinement, the Brazilian prison population is vulnerable to numerous diseases [1], and several studies have sought to identify the main factors associated with diseases that affect this population [2,3,4,5]. However, studies of oral health have been few, and little is known about the oral health status of prisoners in Brazil, although in general, it appears poor [6].

Studies in prisons in India [7], Africa [8], United Kingdom [9], United States [10], Australia [11] and China [12] have shown poor oral health conditions among prisoners, particularly reflected in the high number of lost and untreated decayed teeth. They attribute these conditions to negligence in oral health, drug use, irregular use of dental services [9], and the social exclusion and unemployment experienced by most former prisoners [13].

Due to the significant need for dental treatment required by new arrivals in the prison system and the high number of people sentenced to prison, there is a great demand for dental services in prisons [13]. However, obstacles include unavailability of professionals, low budget for maintenance of equipment and materials, and security issues, which can hinder the routine of appointments [7,14].

Brazil has a government strategy to provide health services to the prison population, the National Plan of Prison Health (PNSSP). States applying for the PNSSP, when qualified, begin to guide their actions toward health promotion and disease prevention, strengthening primary health care activities within prisons, through the insertion of minimum health teams consisting of physician, dentist, nurse, psychologist, social worker, nurse and oral health technicians [15].

Thus, the aim of this study was to evaluate the use of dental services and dental caries experienced among prisoners of prison with a health unit already in operation.

## 2. Methods

### 2.1. Study Population

This cross-sectional study was conducted at the Joao Bosco Carneiro prison located in the municipality of Guarabira, inner state of Paraiba, northeastern Brazil. This prison unit is one of fifteen prisons intended for male imprisonment in the State of Paraiba.

The study population consisted of 206 prisoners, and the non-probabilistic sample of 127 individuals. Individuals under disciplinary isolation or affected by infectious diseases were excluded.

Mediated by a prisoner who for good behavior renders services in the prison, other inmates were invited to participate in the study. Thus, at every shift, five inmates were escorted by prison officers to the room dedicated to the survey.

All willing participants from the prison were included, except those with documented health histories of head and neck radiation therapy or Sjogren’s syndrome or similar exocrine disorders.

### 2.2. Training and Calibration Process

A single gold standard examiner (ALC) managed the calibration process. Agreement analysis used Cohen’s kappa coefficient on a tooth-by-tooth basis. The examiner performed intraoral clinical examination with proper calibration (Kappa = 0.92 and 0.79 to DMFT and CPI indexes, respectively), with the assistance of an annotator properly trained for this function, both wearing aprons to meet general biosecurity standards. For the examination of tooth surfaces, disposable wooden spatulas, gauze, mouth mirrors and properly sterilized millimeter probes (Community Periodontal Index—CPI) were used.

### 2.3. Non Clinical Data Collection and Dental Examinations

The data collection instrument was adapted for the study population based on the Brazilian Oral Health Survey (SB Brazil 2010) using criteria recommended by WHO to asses tooth decay experience [16].

Data were collected through interviews and clinical examination. Initially, data regarding sociodemographic and sentencing profiles of prisoners were obtained, including age, educational level, labor activity prior to arrest, prison time, use of dental services, self-reported dental morbidity, self-perception, and impacts on oral health in the last six months. Then, dental caries experience was evaluated by clinical dental examination [16].

### 2.4. Data Analysis

Statistical Package for Social Sciences (SPSS) software, version 17 assisted with the data organization and analysis. Descriptive statistics obtained absolute and percentage distributions and analytical statistics for statistical calculations. In the bivariate analysis, the median dichotomized the independent variable DMFT, generating two groups (≤21, 22–32). Dependent variables were toothache in the last six months, oral health satisfaction, self-perceived need for dental treatment, discomfort during teeth brushing, pain when drinking hot or cold liquids, difficulty speaking, shame of speaking and smiling, difficulty performing routine activities, and sleeping problems. The level of statistical significance was set at 5% with a confidence interval of 95%. Pearson chi-square statistical tests or Fisher’s exact and Kruskal-Wallis test with comparisons were used.

### 2.5. Ethical Aspects

This study followed ethical guidelines recommended by the Brazilian legislation and was approved by the Human Research Ethics Committee of the State University of Paraiba. All participants and guardians signed the informed consent form.

## 3. Results

The mean age was 28.5 (±7.8) years, with minimum of 18 and maximum of 55 years, with more than half of inmates (52.8%) aged less than 30 years. Most (67.7%) failed to complete primary school and 17.3% were still illiterate. More than half (56.9%) were performing some labor when arrested. The mean prison time was 30.0 (±29.3) months, with 58% of inmates having prison time of 24 months (Table 1).

**Table 1:** Distribution of inmates regarding socio-demographic and sentencing characteristics.

Variable	Frequency		Mean (SD)
	n	%	
Age (years)			28.2 (± 7.82)
18 to 23	40	31.5	
24 to 27	27	21.3	
28 to 33	36	28.3	
34 to 55	24	18.9	
Total	127	100	
Educational level			
Illiterate	22	17.3	
Complete basic education	86	67.7	
Incomplete basic education	4	3.2	
Complete high school	7	5.5	
Incomplete high school	8	6.3	
Total	127	100	
Labor activity prior to arrest			
Yes	77	60.6	
No	50	39.4	
Total	127	100	
Prison time (months)			30.03 (± 29.3)
1–12	44	34.5	
13–24	30	23.5	
25–36	17	13.5	
37–48	13	10.2	
50–180	23	18.3	
Total	127	100	

The mean DMFT index was 19.72 (Table 2). Of components, the decayed component showed the highest average (11.06 ± 5.37), decreasing with increasing age, followed by missing (7.20 ± 7.23), which increased with increasing age, then by filled teeth components (1.46 ± 2.45), whose value did not show any linearity within age groups. The age group from 18 to 23 years had the highest average number of decayed teeth, while for missing and filled components, highest averages were found in the age groups 34–55 years and 24–27 years, respectively.

Regarding the use of dental services, only two inmates reported have never seen a dentist in their lives (Table 3). Of those who had, 80% had their last dental appointment less than one year ago, with most visits occurring in prison (80%). The main reasons for such appointments, in order, were restorative treatment (32%), dental pain (26.4%), and extraction (17.6%).

**Table 2:** Dental caries experience by age group.

	Age Group (Years)				Total	*p* Value
	18–23	24–27	28–33	34–55		
	Mean ± SD	Mean ± SD	Mean ± SD	Mean ± SD	Mean ± SD	
DMFT	16.78 ± 5.80 ^(A)^	20.30 ± 5.86 ^(A)^	20.25 ± 5.83 ^(A)^	23.17 ± 6.39 ^(B)^	19.72 ± 6.29	*p* = 0.001
Decayed	12.62 ± 5.61 ^(A)^	12.04 ± 5.32 ^(B)^	11.19 ± 4.28^(B)^	7.13 ± 4.82 ^(C)^	11.06 ± 5.37	*p* = 0.001
Missing	2.90 ± 3.42	5.93 ± 5.30	7.39 ± 4.81	15.50 ± 9.69	7.20 ± 7.23	*p* < 0.001
Filled	1.25 ± 2.14 ^(A)^	2.33 ± 3.62 ^(B)^	1.67 ± 2.26 ^(B)^	0.54 ± 0.83 ^(B)^	1.46 ± 2.45	*p* = 0.096

^+^ Distinct letters in brackets demonstrate significant differences between corresponding age groups.

**Table 3:** Use of dental services by inmates.

	Frequency	
	n	%
Have you ever been to the dentist before?		
Yes	125	98.4
No	02	1.6
Total	127	100
When was your last dental appointment?		
Less than one year	100	80
Between one and two years	13	10.4
Three or more years	11	8.8
Do not know/No response	01	0.8
Total	125	100
Where was your last visit?		
Prison	100	80
External public service	08	6.4
Private service	13	10.4
Do not know/No response	04	3.2
Total	125	100
Reason for the last appointment?		
Prevention	02	1.6
Pain	33	26.4
Extraction	22	17.6
Restorative treatment	40	32
Others	25	20
Do not know/No response	03	2.4
Total	125	100

There was a significant association of DMFTs between 22 and 32 and oral health dissatisfaction (*p* = 0.002), difficulty speaking (*p* = 0.024), shame of talking (*p* = 0.004) and smiling (*p* < 0.001) (Table 4).

**Table 4:** Relationship between DMFT and tooth pain, satisfaction, self-perception and impact on oral health.

Variable	DMFT				Total		*p*-Value	PR (CI 95%)
	22 to 32		Up to 21					
	n	%	n	%	n	%		
Pain in last 6 months								
Yes	20	39.2	26	36.1	46	37.4	*p* = 0.726	1.09 (0.69–1.72)
No	31	60.8	46	63.9	77	62.6		1.00
Total	51	100.0	72	100.0	123	100.0		
Oral health satisfaction							
Satisfied	17	30.9	42	58.3	59	46.5	*p* = 0.002	1.00
Dissatisfied	38	69.1	30	41.7	68	53.5		1.89 (1.22–2.93)
Total	55	100.0	72	100.0	127	100.0		
Self-perceived need for dental treatment								
Yes	49	89.1	66	91.7	115	90.6	*p* = 0.384	*
No	6	10.9	4	5.6	10	7.9		*
Do not know	-	-	2	2.8	2	1.6		*
Total	55	100.0	72	100.0	127	100.0		
Impacts on oral health								
Difficulty eating								
Yes	19	34.5	16	22.2	35	27.6	*p* = 0.124	1.00
No	36	65.5	56	77.8	92	72.4		1.56 (0.88–2.74)
Total	55	100.0	72	100.0	127	100.0		
Pain when drinking hot or cold liquids **								
Yes	34	47.2	26	51.0	60	48.8	*p* = 0.681	1.08 (0.75–1.55)
No	38	52.8	25	49.0	63	51.2		1.00
Total	72	100.0	51	100.0	123	100.0		
Discomfort when brushing teeth **								
Yes	12	16.7	15	29.4	27	22.0	*P* ^(1)^ = 0.092	1.76 (0.90–3.45)
No	60	83.3	36	70.6	96	78.0		1.00
Total	72	100.0	51	100.0	123	100.0		
Difficulty speaking								
Yes	10	18.2	4	5.6	14	11.0	*p* = 0.024	1.00
No	45	81.8	68	94.4	113	89.0		3.27 (1.08–9.88)
Total	55	100.0	72	100.0	127	100.0		
Shame of speaking								
Yes	14	25.5	5	6.9	19	15.0	*p* = 0.004	1.00
No	41	74.5	67	93.1	108	85.0		3.67 (1.41–9.56)
Total	55	100.0	72	100.0	127	100.0		
Shame of smiling								
Yes	23	41.8	7	9.7	30	23.6	*p* < 0.001	4.30 (1.99–3.29)
No	32	58.2	65	90.3	97	76.4		1.00
Total	55	100.0	72	100.0	127	100.0		
Difficulty in performing daily tasks **								
Yes	2	2.8	2	3.9	4	3.3	*p* = 1.000	*
No	70	97.2	49	96.1	119	96.7		
Total	72	100.0	51	100.0	123	100.0		
Sleeping problems **								
Yes	18	25.0	10	19.6	28	22.8	*p* = 0.482	1.28 (0.64–2.53)
No	54	75.0	41	80.4	95	77.2		1.00
Total	72	100.0	51	100.0	123	100.0		

* Unable to determine due to zero and very low frequencies. ** Not applied to edentulous subjects.

## 4. Discussion

Although many international publications describe oral health status [7,8,9,10,11,12], a review of Brazilian literature reveals no national data or published studies that assess the dental caries of inmates. This is the first study in Brazil to do so, focusing on male inmates.

Given the serious problems faced by the Brazilian prison system such as overcrowding, rebellions, escapes, and drug trafficking, as well as other, obscured issues, the oral health status of prisoners is sometimes dismissed as irrelevant. However, there are high levels of oral diseases among prisoners, the treatment of which can be complex and challenging, due to the urgent demand for dental services [13,17]. In this sense, understanding what is the reality of the oral health conditions among prisoners in order to enlarge and even adequate the dental service provided in the prison environment is critical.

Due to the pioneering nature of this study on local and national levels, we sought a prison whose physical organization would allow faster escort of prisoners and consequently less interference in security: the Joao Bosco Carneiro Penitentiary in Guarabira, Brazil. This prison also stands out for its engagement in the rehabilitation of prisoners through the valuation of prison labor, educational assistance, and encouragement of projects that will help prisoners reintegrate into society.

People aged under 30 are the most numerous among Brazilian prisoners, unlike some countries, whose average prisoner age was between 30 and 41 years [7,9,11,18]. Various theories attempt to explain this; however, educational deficit, unemployment, low income and other socio-economic problems as a consequence of wealth concentration in Brazil are among factors considered inclusive to crime [19].

Low educational level is a common trait among prisoners, which explains the frequency of unemployment and poorly paid jobs prior to arrest [7,19,20]. Little more than half of the prisoners had performed some sort of labor activity not long before being arrested; of those, most occupations required few qualifications and offered little remuneration. Low educational level ends up generating numerous impacts on the lives of individuals: it lowers their income, weakens their (oral) health condition due to limited access to information, and can lead them to crime [21].

The mean DMFT index value found among inmates was quite high (19.72), similar to that of prisoners in Australia [11] (20.4), United Kingdom [17] (14.35), and South Africa [8] (15.45). Dissimilar results were found in studies in India [7] (5.26), the Western Africa [22] (6.5) and Italy [18] (9.8), which shows that caries experience varied considerably within prison populations of different countries, and even within a country. Such differences may be related to methodology used and/or the quality of dental services provided to different population groups inside and outside prisons.

Brazil has a great disparity in prison health care, because since 2003, it established a National Plan of Prison Health (PNSSP) [15], but states have varied in adhesion to the plan even by mid-2012. Even after qualifying for the deployment of health facilities, states’ installation of such facilities can be slow, as in the State of Paraiba, which was qualified for the implementation of 18 health units in prisons in 2008, but by January 2013, had only seven implemented.

Although the real obstacles to state and local administrators’ installation of the facilities are unknown, the health of individuals deprived of their liberty is a right and should be provided to all Brazilian citizens. The PNSSP, based on health promotion and disease prevention, will succeed only if implemented within the prison environment, since care provided outside prisons is virtually restricted to emergency services.

Although Joao Bosco Carneiro Penitentiary has a dental office and a dental surgeon available for the daily care of prisoners, dental records are not used up to the present, which ends up limiting information on the use of dental services and therefore information on caries experience among prisoners. Thus, considering the long prison time of much of the prisoners, it is difficult to precisely understand the real influence of prison time on caries, especially when there is dental care within the prison environment. It is not known how much neglect is from individuals and how much from dental services.

Thus, an initial dental screening of all those who arrive in the prison system is important, to record the main existing oral problems among individuals at the time of arrest. This facilitates the planning of dental prevention and rehabilitation within prisons. Another important aspect is the record of procedures performed during prison, which make it possible to evaluate the real effectiveness of dental services provided during incarceration, as well as the evolution of patients’ dental self-care.

Evaluating dental services is critical, as it appears that there was a greater use of dental services in the past year, mostly within prisons, but satisfactory results were not obtained because although restorative treatment was the reason for the last dental visit for many prisoners, low numbers of filled teeth and high numbers of missing and untreated teeth components were found in all age groups, suggesting that the use of dental services do not always lead to satisfactory oral health.

Another condition that has increased the use of dental services within the prison environment is dental pain, as observed in this and other studies [7,9]. Such a condition has been justified by the fact that drug use, very common among prison inmates before arrest, suppressed pain and then was prohibited in prison, reducing the pain threshold and causing greater demand for dental services [13]. However, dental patients treated as emergency cases return for new appointments more often and receive more surgical procedures, representing a punctual solution for problems, and therefore present more long-term needs of accumulated treatment, beginning a series of emergency services that harm their oral health [21].

Considering the impacts on oral health—such as oral health dissatisfaction, difficulty speaking, and shame of smiling and talking—found among prisoners with high DMFT values, dental diseases can directly or indirectly interfere in the normal and desirable functioning of individuals, including functional, psychological, and even social aspects. Efforts must be made to reestablish functional and comfortable dentition, and an appearance that will enable these individuals to carry out their daily activities without physical, psychological, or social disorders, considering that the oral health rehabilitation of individuals deprived of their liberty can have a key role in social reintegration.

Some limitations should be considered. The cross-sectional design limits cause and effect inferences, only demonstrating the presence or absence of associations. Another limitation relates to the sample selection, which was for convenience due to difficulties the prison management reported in the designation of police escort to accompany prisoners during examinations. The question of whether the study results have external validity is often a matter of judgment that depends on the study setting, the characteristics of the participants, the exposures examined, and the outcomes assessed [23]. The population group examined is assumed to be relevant for comparisons of both Brazilian prisoners and other prisoner nationalities.

Although the implementation of the Oral Health Policy in Brazil has considerably improved the access to dental care services, a large portion of the population is still marked by mutilating procedures and without access to prosthetic rehabilitation [24]. Although the questions on quality of life have not been formally validated, they have been used by Brazilian Ministry of Health in national oral health surveys.

Regarding the profile of prisoners found in this study and their caries, this group of individuals was marked by hardship in many areas of life, including lack of opportunity, which may have led to their involvement in illicit activities. Since one cannot change the past, we must seek to improve the present, and considering these individuals’ imprisonment and idleness, there is an opportunity to encourage them to study, work, and recover their health, including oral health, as a way to return to society in a more dignified, less stigmatized, and less prejudiced way.

## 5. Conclusions

Most prisoners used the dental services provided by the prison. Although restorative treatment has been the main reason for the use of dental services, “decayed” and “missing” components contributed to the high mean DMFT index.
